# Impact of age for overall survival in head and neck sarcoma

**DOI:** 10.1097/MD.0000000000032966

**Published:** 2023-02-17

**Authors:** Hidenori Suzuki, Gaku Takano, Satoshi Tsukushi, Masashi Ando, Yasushi Yatabe, Takeshi Kodaira, Daisuke Nishikawa, Shintaro Beppu, Yasuhisa Hasegawa, Nobuhiro Hanai

**Affiliations:** a Department of Head and Neck Surgery, Aichi Cancer Center Hospital, Nagoya, Japan; b Department of Otolaryngology-Head and Neck Surgery, Nagoya City University Graduate School of Medical Science, Nagoya, Japan; c Department of Orthopedic Surgery, Aichi Cancer Center Hospital, Nagoya, Japan; d Department of Clinical Oncology, Aichi Cancer Center Hospital, Nagoya, Japan; e Department of Pathology and Molecular Diagnostics, Aichi Cancer Center Hospital, Nagoya, Japan; f Department of Radiation Oncology, Aichi Cancer Center Hospital, Nagoya, Japan; g Department of Head and Neck Surgery, Asahi University, Gifu, Japan.

**Keywords:** age, chemotherapy, head and neck sarcoma, overall survival, predictor

## Abstract

The purpose to the present study is to research the association between age at surgery and survival outcomes of patients with sarcoma in head and neck. Twenty-six patients with head and neck sarcoma who underwent by surgery from 2003 to 2017 were enrolled in the present observation study. Patients who did not undergo chemotherapy were significantly older age at surgery by Mann–Whitney *U* test. Fifty-five was the cutoff age that predicted death by receiver operating curve analysis. Shorter survival rates of overall, disease-specific, local recurrence-free and disease-free were associated with older age by log-rank test. Age (≥55 years/<55 years) was correlated with shorter overall survival by multivariate analysis of Cox’s proportional hazards model adjusting with chemotherapy (absence/presence). In conclusion, older age predicts worse overall survival in head and neck sarcoma.

## 1. Introduction

Head and neck sarcoma (HNS) is a rare malignancy, with more than 50 pathological subtypes, accounting for approximately 1% of all head and neck malignancies.^[1,2]^ Surgery is the main treatment for patients who are newly diagnosed with HNS, while the efficacy of chemotherapy or radiotherapy remains debatable for all pathological subtypes of HNS.^[1–3]^ The predictors for survival outcomes of HNS, such as overall survival, have been investigated by several previous studies.^[1–9]^

The age of patients with various types of malignant tumors, including HNS, were evaluated as an important predictor to guide management.^[4,5,10]^ We also investigated the significant association between shorter overall survival of thyroid carcinoma and age ≥65 years at salvage surgery based on receiver operating curve (ROC) analysis.^[10]^ Several previous studies on HNS have shown that older age have worse survival outcomes.^[4,5]^ The association between age and survival outcomes in HNS has yet to be fully investigated due to rare tumor as well as lack of prospective randomized trials,^[6]^ and there is rationale to accumulate further data for HNS.

Therefore, in the current study, we investigated the possible association between the age and survival outcomes of patients with HNS.

## 2. Materials and methods

### 2.1. Patient selection

Between September 2003 and March 2017, 29 patients with HNS underwent surgery with curative intent at Aichi Cancer Center Hospital. Among the 29 patients, 3 who underwent salvage surgery for recurrent disease were excluded, and 26 patients were finally enrolled. The study also included one patient with epithelioid haemangioendothelioma who was mentioned as a case report.^[11]^ This retrospective study was approved by our institution’s review board (receipt number: 2017-1-052) according to the Declaration of Helsinki on ethics and medical protocol.

### 2.2. Clinicopathological parameters

The age of the patients at the time of surgery was recorded. The tumor-node-metastasis (TNM) classification based on the Union for International Cancer Control was determined through physical examination, endoscopy, cervical magnetic resonance imaging and/or computed tomography (CT), and positron-emission tomography using 18F-2-fluorodeoxyglucose combined with CT if possible. The patients underwent surgery for curative intent, with and without tissue flap reconstruction in 20 and 6 patients, respectively. Pathological reports were prepared by two experienced pathologists who compiled all available reports. The surgical margins from the pathological reports were divided into positive and negative as described previously.^[12]^ The pathology and tumor location were defined using the eighth edition of the American Joint Committee on Cancer’s Cancer Staging Manual, as previously described.^[10,13]^ Pre- and postoperative therapies such as chemotherapy and radiotherapy were recommended after consulting with orthopedic and/or medical oncologists, as well as the pathological report; for example, a positive surgical margin. Systemic chemotherapy was administered as the front line regime as follows: A combination of doxorubicin (DOX) and ifosfamide (IFM) or DOX for non-round cell tumors; a combination of vincristine (VCR), actinomycin D, and cyclophosphamide (CPA) for rhabdomyosarcoma; a combination of VCR, DOX, and CPA or the combination of etoposide and IFM for Ewing’s sarcoma/peripheral primitive neuroectodermal tumor; and a combination of DOX and cisplatin, a combination of IFM and etoposide, or high-dose methotrexate for osteosarcoma. We suitably adjusted the dose and cycles of chemotherapy based on the patient’s age and adverse effects. Two patients were pathologically diagnosed with no residual tumor cells on the surgical specimens following preoperative chemotherapy; one patient with Ewing sarcoma and another patient with rhabdomyosarcoma. These patients were diagnosed by pretreatment pathological examination, underwent three cycles of DOX, VCR, IFM, actinomycin D and four cycles of VCR, actinomycine D, CPA, respectively. Salvage surgery for early locoregional recurrence was performed on the basis of biopsy results and specific tests such as CT by follow up at outpatient clinic after treatment completion. The median follow-up duration from the time of surgery for the 13 patients who were still alive and the 13 who died was 1869 days (range, 299–6096 days) and 524 days (range, 171–1608 days), respectively. The clinicopathological parameters are shown in Table 1.

**Table 1:** Clinicopathological parameters of 26 patients with head and neck sarcomas.

Parameter	Number
Age	
49≥/<49	13/13
Sex	
Male/female	17/9
Pathology	
Osteosarcoma	8
Rhabdomyosarcoma	6
Undifferentiated pleomorphic sarcoma	3
Chondrosarcoma	2
Ewing sarcoma	2
Hemangiopericytoma	1
Adult fibrosarcoma	1
Extraskeletal osteosarcoma	1
Epithelioid hemangioendothelioma	1
Epithelioid malignant peripheral nerve sheath tumor	1
Site	
Bone/soft tissue	12/14
pT	
pT3/pT2/pT1/pT0	8/6/10/2
pN	
pN1/pN0/pNX	2/14/10
Surgical margin	
Positive/negative	5/21
Histological grade	
Grade3/Grade2/Grade1/GradeX	5/2/2/17
Location	
Mandible	17
Cheek mucosa	4
Tongue	2
Nasal cavity	2
C41.0	2
C49.0	2
Maxillary sinus	1
Larynx, NOS	1
Parotid gland	1
C47.0	1
Radiotherapy	
Absence/presence	4/22
Chemotherapy	
Absence/presence	13/13
Last contact	
Death/alive	13/13

C41.0 = bones of the skull, face, and associated joints, C49.0 = connective, subcutaneous, and soft tissues of the head, face, and neck, C47.0 = peripheral nerves and autonomic nervous system of the head, face, and neck.

### 2.3. Data analysis

Data analyses were statistically performed using JMP software package (version 9; SAS, Cary, NC), and *P* values < .05 were considered significantly significant. The association between age and clinicopathological parameters was assessed by Mann–Whitney *U* test. The optimal cutoff values for age to predict death with area under the curve (AUC), sensitivity and 1-specificity were estimated by using ROC analysis, as reported previously.^[10]^ The survival time (days from surgery to last contact or a target event) was computed by the Kaplan–Meier method. The target events were death for overall survival; death due to HNS for disease-specific survival; local recurrence for local recurrence-free survival; regional recurrence for regional recurrence-free survival; distant metastasis for distant metastasis-free survival; and local or regional recurrence, and distant metastasis for disease-free survival. Overall survival and age for continuous variables per 1 age was assessed by Cox proportional hazard model with hazard ratio (HR) and 95% confidence interval (95% CI). Univariate survival analysis for the differences between two groups based on age (≥55 years/<55 years) and the absence/presence of chemotherapy were compared by log-rank test. Smoking history was 14 patients with the presence and 12 patients with the absence, and the association between smoking history and survival outcomes was compared by log-rank test. Multivariate survival analysis adjusted for age (≥55 years/<55 years) and the absence/presence of chemotherapy was performed using the Cox proportional hazards model.

## 3. Results

### 3.1. Clinical course

Among all patients, 11, 9, 2, and 12 patients died as a result of HNS, local recurrence, regional recurrence, and distant metastasis, respectively. The median durations from surgery until death from HNS, local recurrence, regional recurrence, and distant metastasis were 488 days (range, 171–905 days), 101 days (range, 56–708 days), 382.5 days (range, 382–383 days), and 320 days (range, 56–554 days), respectively. The 4-year survival rates of overall, disease-specific, local recurrence-free, regional recurrence-free, distant metastasis-free, and disease-free survival were 52.3%, 56.3%, 64.6%, 90.5%, 52.6%, and 46.2%, respectively.

### 3.2. Age and clinicopathological parameters

**Figure 1.**
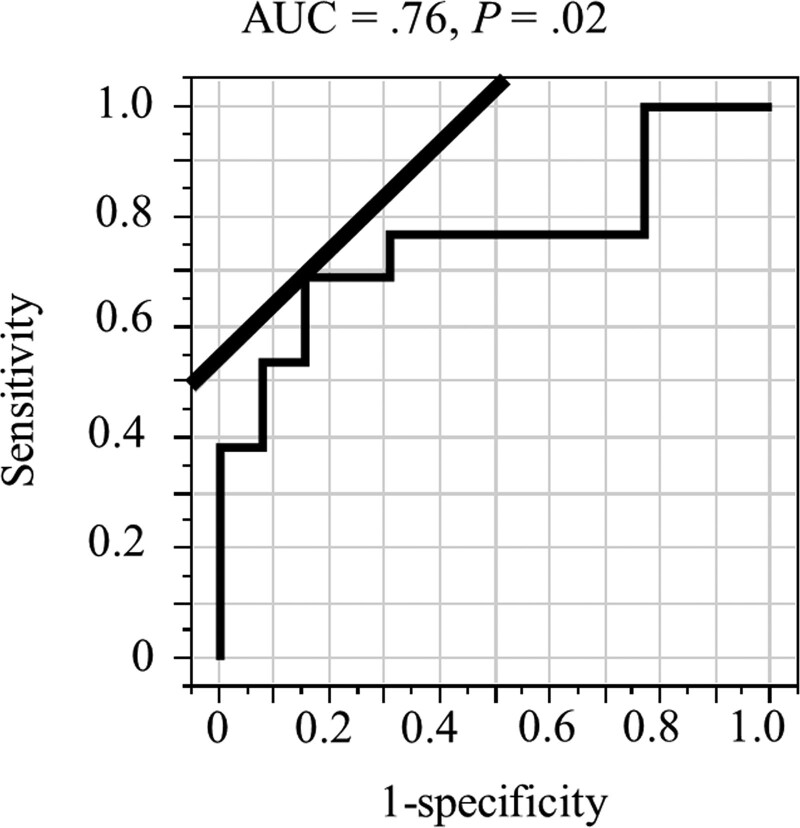
The Mann–Whitney *U* test showed that significant relationship between death and alive based on age at surgery (*P =* .02).

The association between age and clinicopathological parameters is presented in Table 2. Patients who did not undergo chemotherapy were significantly older age at the surgery than those who did with (*P <* .01). Association between alive and death according to age at surgery is shown in Figure 1. Patients who had died by the last contact were significantly older at the time surgery than those who survived (*P =* .02).

**Table 2:** Associations between age and clinicopathological parameters in 26 patients of head and neck sarcomas.

Parameter	Number	Age (mean ± standard deviation)	*P* value*
Sex			
Male/female	17/9	44.7 ± 21.4/52.4 ± 20.2	.33
Pathology			
Osteosarcoma/others	8/18	45.8 ± 21.2/48.1 ± 21.4	.78
Rhabdomyosarcoma/others	6/20	35.2 ± 19.0/51.1 ± 20.5	.09
Undifferentiated pleomorphic sarcoma/others	3/23	50.7 ± 19.0/47.0 ± 21.6	.84
Chondrosarcoma/others	2/24	59.5 ± 21.9/46.4 ± 21.1	.36
Ewing sarcoma/others	2/24	26.5 ± 20.5/49.1 ± 20.5	.12
Site			
Bone/soft tissue	12/14	44.1 ± 22.0/50.2 ± 20.5	.49
pT			
pT3/pT0-2	8/18	58.1 ± 20.2/42.6 ± 20.0	.07
pN			
pN1/pN0-NX	2/24	47.0 ± 33.9/47.4 ± 20.7	.92
Surgical margin			
Positive/negative	5/21	55.6 ± 19.4/45.4 ± 21.3	.34
Histological grade			
Grade1, GradeX/Grade2-3	19/7	49.3 ± 21.5/42.1 ± 19.9	.43
Location			
Mandible/others	17/9	51.1 ± 21.2/40.3 ± 19.7	.24
Cheek mucosa/others	4/22	48.5 ± 23.7/47.2 ± 21.0	.89
Tongue/others	2/24	28.5 ± 17.7/49.0 ± 20.8	.15
Nasal cavity/others	2/24	43.5 ± 29.0/47.7 ± 21.0	.85
C41.0/others	2/24	45.5 ± 33.2/47.5 ± 20.7	.81
C49.0/others	2/24	48.5 ± 10.6/47.3 ± 21.7	1.00
Radiotherapy			
Absence/presence	4/22	47.0 ± 20.9/49.8 ± 24.5	.89
Chemotherapy			
Absence/presence	13/13	59.2 ± 17.8/35.5 ± 17.2	<.01
Last contact			
Death/alive	13/13	56.2 ± 21.2/38.5 ± 17.2	.02

C41.0 = bones of the skull, face, and associated joins, C49.0 = connective, subcutaneous, and soft tissues of the head, face, and neck.

* Mann–Whitney *U* test.

### 3.3. Cutoff value of age

**Figure 2.**
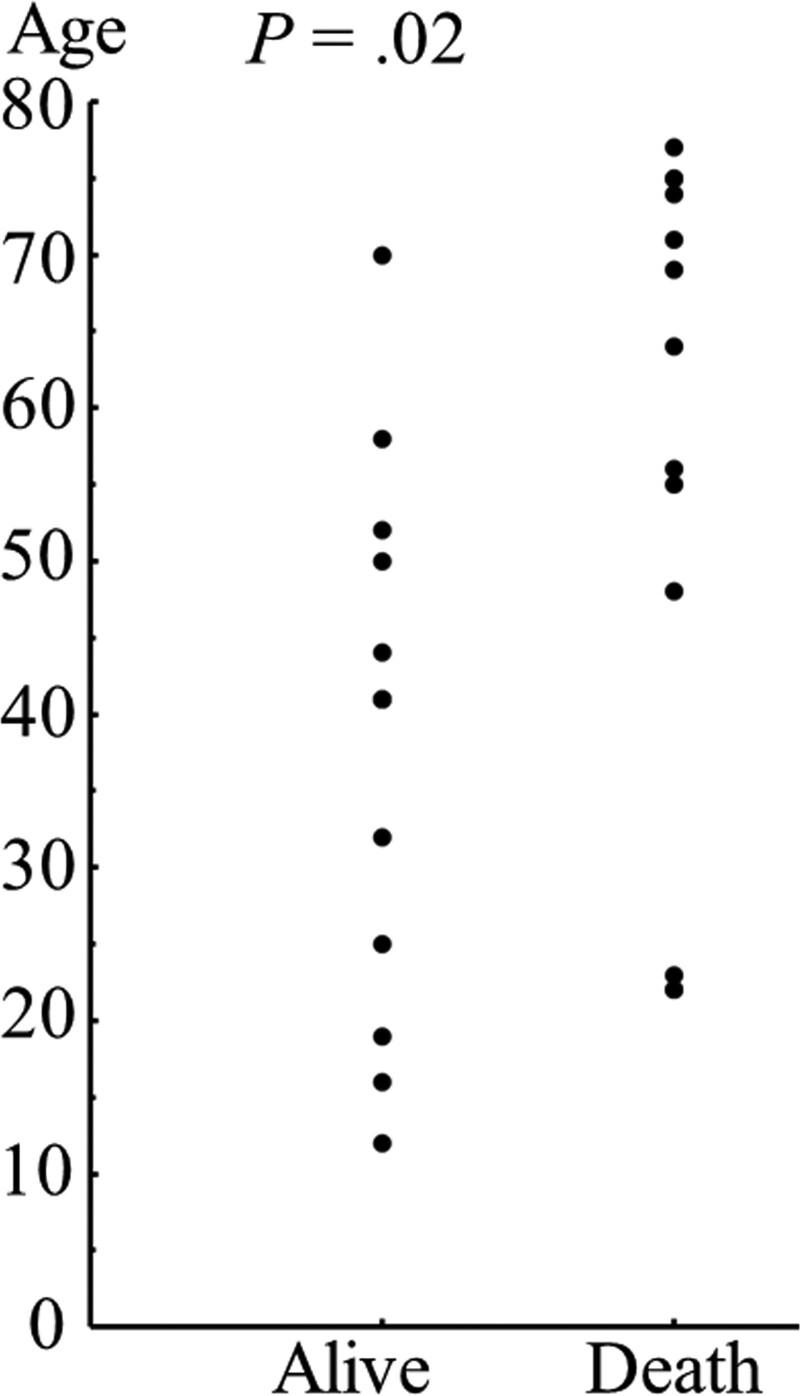
Receiver operating curve of the age at surgery for predicting death in 26 patients with head and neck sarcoma with the straight line at a 45° angle tangent (*P* = .02, AUC = .76). AUC = area under the curve.

The ROC, sensitivity, 1-specificity, and AUC of the ROC are presented in Figure 2. The optimal cutoff value of age for predicting death was 55 years old (AUC = .76, *P* = .02). The number of patients with age ≥ 55 years and age < 55 years were 11 and 15, respectively.

### 3.4. Univariate survival analysis

**Figure 3.**
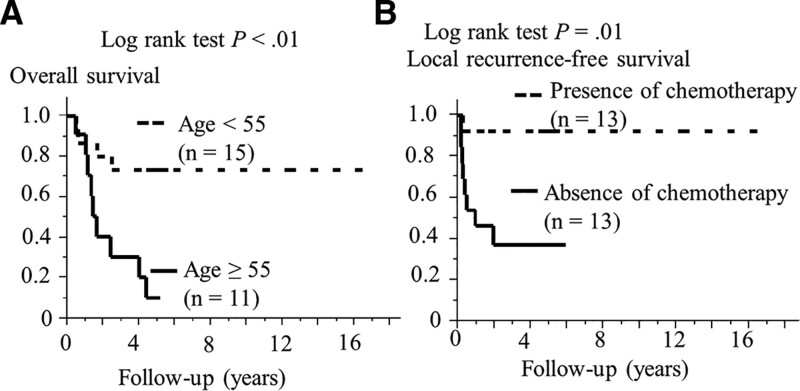
Kaplan–Meier survival curves of 26 patients. The log-rank test showed that (A) age ≥55 years at surgery was significantly associated with shorter overall survival than age <55 years (*P <* .01), and that (B) the absence of chemotherapy was related to shorter local recurrence-free survival than the presence of chemotherapy (*P =* .02).

There was a significant association between overall survival and age for continuous variables (HR: 1.03, 95% CI: 1.01–1.07, *P* = .02). The survival results of univariate and multivariate analyses are presented in Table 3, and the Kaplan–Meier curves are shown in Figure 3. In univariate analysis, patients with age ≥55 years in comparison to those of age <55 years had significantly worse overall (*P* < .01), disease-specific (*P* = .03), local recurrence-free (*P* < .05), and disease-free (*P* = .03) survival. Moreover, log-rank test showed that patients who did not undergo chemotherapy had shorter overall (*P =* .03), local recurrence-free (*P =* .01), and disease-free (*P* = .01) survival compared to those who did. Smoking history was not significantly associated with overall (*P =* .40), disease-specific (*P =* .17), local recurrence-free (*P =* .95), regional recurrence-free (*P =* .97), distant metastasis-free (*P =* .30), and disease-free (*P =* .66) survival.

**Table 3:** Survival analyses for 26 head and neck sarcomas.

Survival	Age (≥55 years/<55 years)				Chemotherapy (absence/presence)			
	Univariate		Multivariate		Univariate		Multivariate	
	*P* value*	HR	95% CI	*P* value†	*P* value*	HR	95% CI	*P* value†
Overall	<.01	4.09	1.15–17.1	.03	.03	1.88	0.54–7.75	.33
Disease-specific	.03	3.00	0.77–13.3	.12	.13	1.52	0.39–6.66	.55
Local recurrence-free	<.05	1.88	0.47–9.58	.38	.01	8.46	1.37–164	.02
Regional recurrence-free	.75	1.44	0.04–51.2	.83	.81	1.16	0.03–41.3	.93
Distant metastasis-free	.07	1.88	0.51–7.60	.34	.05	2.31	0.60–10.0	.23
Disease-free survival	.03	1.87	0.59–6.41	.29	.01	3.18	0.93–12.7	.07

95% CI = 95% confidence interval, HR = hazard ratio.

* Log-rank test.

† Cox proportional hazards model.

### 3.5. Multivariate survival analysis

In multivariate analysis, age (≥55 years/<55 years) was correlated with poor overall survival (HR: 4.09, 95% CI: 1.15–17.1, *P* = .03), while the absence/presence of chemotherapy was associated with poor local recurrence-free survival (HR: 8.46, 95% CI: 1.37–164, *P* = .02).

## 4. Discussion

The univariate and multivariate analyses of 26 patients with HNS showed significant correlation between age ≥ 55 years and shorter overall survival, as well as a significant relationship between the absence of chemotherapy and worse local recurrence-free survival.

HNSs have been divided into those of the bone and those of the soft tissue.^[1,2]^ Although the TNM staging system has been broadly accepted as a useful predictor for various malignant tumors, there remains a lack of clarification for soft tissue sarcomas of the head and neck in the staging system outlined in the eighth edition of the Union for International Cancer Control TNM classification.^[13]^ Previous studies that included a larger cohort of 12,755 patients mentioned that further data accumulation for predictors of HNS due to rare tumors and various histological subtypes is needed.^[1–3]^ Indeed, Ketabchi et al^[7]^ had reported that the surgical margin was a survival predictor for 25 patients with HNS.

Although no significant association between age and survival was reported,^[14]^ age has been shown to predict overall survival of patients with HNS in both single and multi-institution studies.^[4,5]^ Indeed, single institution study of 186 patients with HNS showed close relationship between age and overall survival.^[4]^ Moreover, a multi-institution study of 214 patients with HNS from the Society of Head and Neck Surgeons showed that age < 18 years was significant predictor for overall survival.^[5]^ Therefore, the finding of a significant relationship between higher age and shorter overall survival in the present study is in good agreement with the findings of previous studies.^[4,5]^ Although the two previous studies did not mention the method used to determine the cutoff ages, the present study used ROC analysis for this purpose.^[4,5]^

Although the value of chemotherapy for HNS is an ongoing debate,^[1,2]^ single and multi-institution studies have shown that chemotherapy improves the survival outcomes for patients with sarcoma, including those with HNS.^[8,9,15]^ Boon et al^[8]^ reported the absence of chemotherapy led to higher local recurrence in 77 patients with osteosarcoma in head and neck. Furthermore, Chen et al^[9]^ also showed that the absence of chemotherapy was associated with shorter overall survival in 157 patients with head and neck osteosarcoma. The European Organization for Research and Treatment of Cancer-Soft Tissue and Sarcoma group showed that IFM-based chemotherapy was an independent predictor in patients with advanced soft tissue sarcoma.^[15]^ The findings of a significant relationship between the absence of chemotherapy and shorter local recurrence-free survival in the present study is consistent with the results of previous reports.^[8,9,15]^

Because patients with age ≥ 55 years had significantly shorter disease-specific survival than those with age < 55 years in the present study, we considered that the significant association between older age and worse overall survival in HNS was caused by a shorter disease-specific survival.

The present study was limited by several bias including its retrospective design, heterogeneity, and relatively small number of subjects. Although the multivariate analysis of the present study showed a significant association between age and overall survival after adjusting for the absence/presence of chemotherapy, a larger cohort of subjects is necessary to determine whether age could be used as a useful predictor with the generalizability to guide treatment.

In conclusion, we demonstrated that age in HNS is significantly correlated with overall survival.

## Author contributions

**Conceptualization:** Hidenori Suzuki.

**Data curation:** Gaku Takano, Satoshi Tsukushi, Yasushi Yatabe, Takeshi Kodaira, Daisuke Nishikawa, Shintaro Beppu, Yasuhisa Hasegawa, Nobuhiro Hanai.

**Formal analysis:** Hidenori Suzuki.

**Writing – original draft:** Hidenori Suzuki, Masashi Ando.

**Writing – review & editing:** Hidenori Suzuki, Gaku Takano, Satoshi Tsukushi, Masashi Ando, Yasushi Yatabe, Takeshi Kodaira, Daisuke Nishikawa, Shintaro Beppu, Yasuhisa Hasegawa, Nobuhiro Hanai.
